# Myelin sheath injury and repairment after subarachnoid hemorrhage

**DOI:** 10.3389/fphar.2023.1145605

**Published:** 2023-04-03

**Authors:** Mao Chen, Peiwen Guo, Xufang Ru, Yujie Chen, Shilun Zuo, Hua Feng

**Affiliations:** ^1^ Department of Neurology, Xinqiao Hospital, Third Military Medical University (Army Medical University), Chongqing, China; ^2^ Department of Neurosurgery and State Key Laboratory of Trauma, Burn and Combined Injury, Southwest Hospital, Third Military Medical University (Army Medical University), Chongqing, China; ^3^ Chongqing Key Laboratory of Precision Neuromedicine and Neuroregenaration, Southwest Hospital, Third Military Medical University (Army Medical University), Chongqing, China; ^4^ Chongqing Clinical Research Center for Neurosurgery, Southwest Hospital, Third Military Medical University (Army Medical University), Chongqing, China

**Keywords:** subarachnoid hemorrhage, white matter, myelin sheath, oligodendrocyte, secondary brain injury

## Abstract

Subarachnoid hemorrhage (SAH) can lead to damage to the myelin sheath in white matter. Through classification and analysis of relevant research results, the discussion in this paper provides a deeper understanding of the spatiotemporal change characteristics, pathophysiological mechanisms and treatment strategies of myelin sheath injury after SAH. The research progress for this condition was also systematically reviewed and compared related to myelin sheath in other fields. Serious deficiencies were identified in the research on myelin sheath injury and treatment after SAH. It is necessary to focus on the overall situation and actively explore different treatment methods based on the spatiotemporal changes in the characteristics of the myelin sheath, as well as the initiation, intersection and common action point of the pathophysiological mechanism, to finally achieve accurate treatment. We hope that this article can help researchers in this field to further clarify the challenges and opportunities in the current research on myelin sheath injury and treatment after SAH.

## 1 Introduction

Subarachnoid hemorrhage (SAH) is one of the most common diseases and normally causes a high mortality rate, 80% of which is caused by intracranial aneurysm rupture. With the rapid development of treatment methods (especially interventional techniques), the survival rate of patients with aneurysmal SAH has reached approximately 65%. However, studies have noted that in the United States, the in-hospital mortality of patients with ruptured aneurysms has not significantly decreased (from 13.7% in 2006% to 13.1% in 2018) (Wahood et al., 2022). Moreover, many survivors suffer from severe neurological deficits (Neifert et al., 2021), thus causing significant negative impacts on not only themselves but also their families and society.

Research on the mechanism of neurological deficits after SAH has been performed for decades, and these deficits have yet to be fully clarified. A large number of studies have suggested that white matter injury plays an important role. It is well known that white matter is mainly composed of axons of neurons and myelin sheaths of oligodendrocytes (OLs). As an important functional unit of OLs, the myelin sheath deserves special attention. A previous study found that the model animals demonstrated myelin sheath injury 4 h after SAH, and active treatment was able to improve the neurological deficits (Toyota et al., 2019). This scenario indicated that myelin sheath injury occurs earlier after SAH, and active treatment is of great significance.

In this study, the spatiotemporal change characteristics, pathophysiological mechanisms and current treatment strategies of myelin sheath injury after SAH are described in detail. Subsequently, through a systematic review and comparative analysis of the important achievements of myelin sheath research in adjacent fields, the shortcomings of the current research were identified. Finally, new research ideas can be adopted in future research on myelin sheath injury and treatment after SAH.

## 2 Research status of myelin sheath injury and treatment after SAH

### 2.1 The spatiotemporal change characteristics of myelin sheath injury after SAH

The myelin sheath was shown to be rapidly damaged and slowly recovered after SAH. Animal studies have shown that myelin basic protein (MBP) content in the corpus callosum can significantly decrease at 3 h after SAH (4). Four hours after SAH, 87.5% (35/40) of experimental animals were found to have T2-hyperintensity in white matter (which was closely related to degradation of myelin protein MBP) *via* cranial MRI (3). Moreover, at 72 h after SAH, the MBP protein content in the corpus callosum decreased to the lowest value (Peng et al., 2019) and then slowly recovered; however, it was still lower than that in the control group at 1 week (Wang et al., 2022). It is well known that the integrity of the myelin sheath is closely related to neurological function. A previous study found that the mNSS score at 2 weeks of SAH experimental animals (Li et al., 2018) and the spatial learning memory and cognitive function at 5 weeks were considerably lower than those of the control group (Wang et al., 2022).

The location of myelin sheath injury is closely related to the puncture point of SAH but is not limited to the area around the puncture point. When considering the threading method as an example, the total incidence of T2-hyperintensity in the white matter was 87.5% (35/40), of which 57% (20/35) were unilateral [of only 10% (2/20) on the same side as the puncture point, 90% (18/20) were contralateral], and 43% (15/35) were bilateral at 4 h after SAH (3). Nevertheless, the collective white matter of the experimental animals exhibited T2-hyperintensity after 24 h of SAH (7).

In summary, SAH-induced myelin sheath injury had obvious spatiotemporal change characteristics, which provides experimental evidence and a research basis for accurately maintaining the integrity of myelin sheath structure and function.

### 2.2 Pathophysiological mechanism involved in myelin sheath injury caused by SAH

**FIGURE 1:**
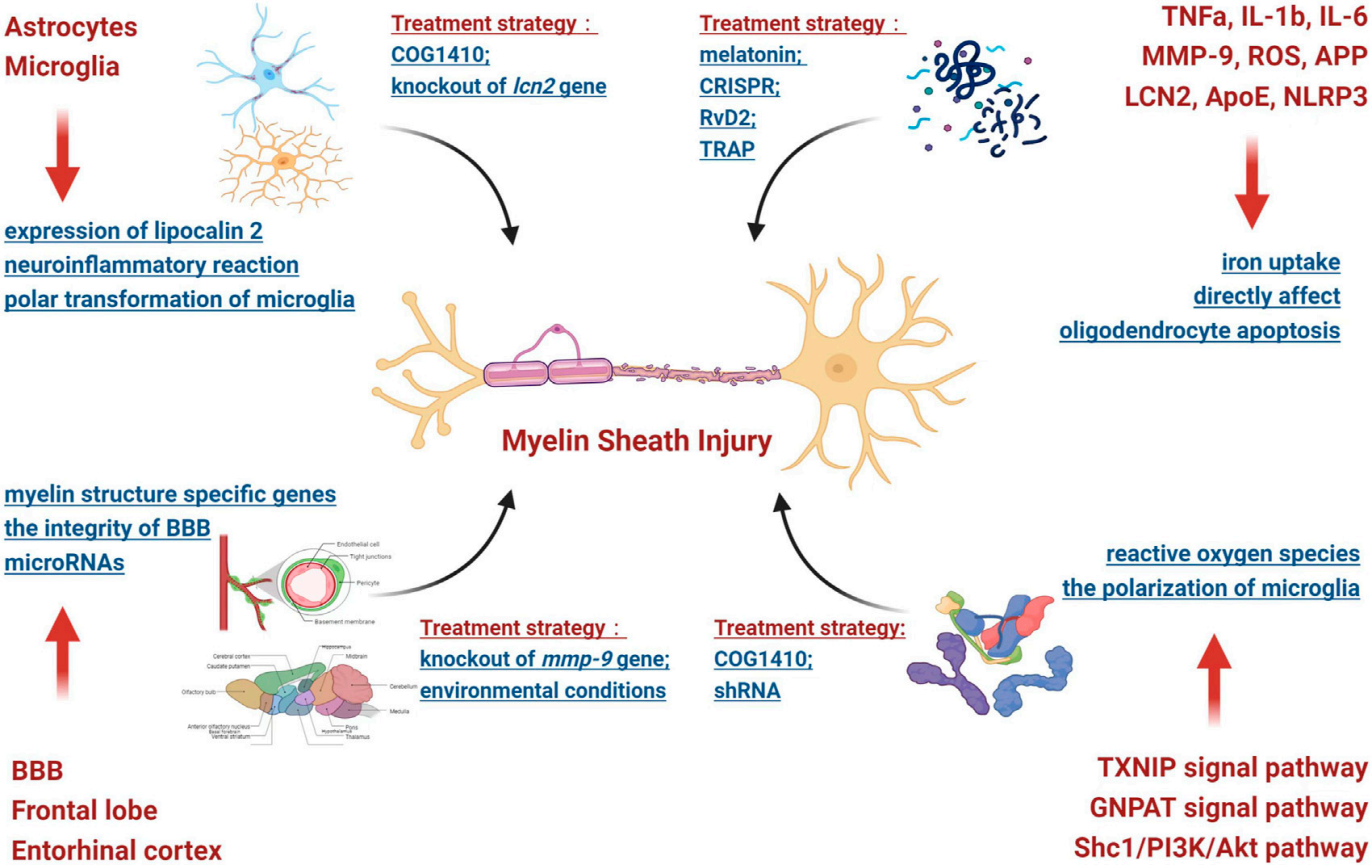
Pathophysiological mechanism involved in myelin sheath injury.

SAH can cause white matter injury through increased intracranial pressure, the outflow of blood components and their metabolites and microcirculation disorders, among causes. In addition, studies have shown that cerebral ischemia and hypoxia caused by microcirculation disorders can induce oxidative stress, excitotoxicity and neuroinflammatory reactions, which can easily damage the myelin sheath. Therefore, under the influence of the abovementioned pathogenic factors, SAH can affect the structural and functional integrity of the myelin sheath through a variety of pathophysiological mechanisms. Thus, this scenario was analyzed as follows (Figure 1).

#### 2.2.1 Involved brain regions

The first analyzed brain area was the frontal lobe. Frontal lobe injury can easily lead to cognitive dysfunction. Moreover, SAH can have an influence on the expression of microRNAs (such as mir-132, mir-134 and mir-138, etc.) in rat frontal lobe brain tissue and subsequently affect the stability of the myelin sheath structure (Ergen et al., 2021). The second analyzed brain area was the entorhinal cortex. Through a systematic RNA sequencing screen of the hippocampus of mice, it was found that SAH-induced damage to the entorhinal cortex may remotely trigger specific hippocampal responses, thereby significantly reducing the expression of myelin structure-specific genes (such as *pllp, mbp, mal, mag, cntnap1, plp1, mog* and *mobp*, among other genes), thus leading to injury (Regnier-Golanov et al., 2021). Eventually, the blood‒brain barrier (BBB) in white matter was identified as being a factor. SAH can damage the integrity of the BBB *via* secondary microcirculation disorders. Therefore, blood components and their metabolites can enter white matter in large quantities and damage the structure and function of the myelin sheath through direct or indirect effects. For example, matrix metalloproteinases (MMPs), especially MMP-9, can destroy the integrity of the BBB in the white matter and myelin sheath; in addition, the inhibition of its expression can produce a protective effect (Egashira et al., 2015).

#### 2.2.2 Involved brain cells

At present, research has mainly focused on astrocytes and microglia. Astrocytes are widely distributed in white matter and can have a rapid impact on the myelin sheath. SAH may cause the rapid expression of lipocalin 2 (LCN2) in the astrocytes located in white matter (which is believed to be the pathological basis of MRI white matter T2-hyperintensity) and subsequently damage the myelin sheath (Toyota et al., 2019). Obviously, astrocytes play an important role in the occurrence and development of myelin sheath injury after SAH. Microglia are closely related to neuroinflammatory reactions, and the persistent inflammatory microenvironment induced by microglia may be the potential mechanism of persistent injury of the myelin sheath after SAH in mice (Wu et al., 2017). In addition, abnormal polar transformation (M1/M2) of microglia also plays an important role in myelin sheath injury. By modulating M2 microglial polarization, it was shown to be helpful in alleviating the myelin sheath injury caused by SAH (4).

#### 2.2.3 Involved molecules and signal pathways

Previous studies have noted that the molecules that cause myelin sheath injury mainly fall into the following three categories after SAH: inflammatory molecules, lipid molecules and apoptotic molecules. For example, when considering inflammatory molecules, MMP-9, ROS, amyloid precursor protein (APP), TNFα, IL-1β, IL-6 and other molecules can directly affect the structure and function of the myelin sheath (Simard et al., 2012; Egashira et al., 2015; Xu et al., 2020). Moreover, ROS are involved in the TXNIP and GNPAT signaling pathways (Xu et al., 2020). When considering lipid molecules, lipoprotein 2 (LCN 2) plays an essential role in myelin sheath injury after SAH (3, 7, 14). The possible mechanism involves the binding of this molecule to its receptor and participation in iron uptake after intracerebral hemorrhage, thus leading to cell injury (Ni et al., 2015) and impaired oligodendrocyte precursor cell differentiation (Li et al., 2022). Endogenous apolipoprotein E (Apo E) may also affect the polarization of microglia through the Shc1/PI3K/Akt signaling pathway and participate in myelin sheath damage (Peng et al., 2019). For apoptotic molecules, NOD-like receptor thermal protein domain associated protein 3 (NLRP3) can destroy the myelin sheath by promoting oligodendrocyte apoptosis (Liu et al., 2020a).

The abovementioned analysis demonstrated that myelin sheath injury after SAH was the result of the combined action of multiple pathophysiological mechanisms involving different brain tissues, brain cells, molecules and signaling pathways. These factors were combined together and interacted, thus making this scenario extremely complex.

### 2.3 Treatment strategy for myelin sheath injury caused by SAH

The degree of myelin sheath injury was shown to be positively correlated with the severity of SAH. As proof, a previous study found that a higher SAH score corresponded to a larger volume of T2-hyperintensity in white matter (Egashira et al., 2014). Accordingly, the treatment of myelin sheath injury plays a vital role in improving secondary neurological deficits after SAH and has become a research hotspot in this field. At present, there are three main research directions (Table 1).

**TABLE 1:** Summary of therapeutic studies on myelin sheath injury caused by experimental SAH.

Category	Name	Dosage	Main outcome	Ref
Endogenous Proteins or Molecules	Heparin	10 U/kg/h	Attenuate adverse neuroinflammatory effects	Simard et al. (2012)
	Melatonin	50 mg/kg	Attenuating apoptosis in oligodendrocytes	Liu et al. (2020a)
Exogenous Proteins or Molecules	COG1410	0.6 mg/kg	Modulating microglial polarization	Peng et al. (2019)
	RvD2	0.9 μg/kg	1. Elevating myelin basic protein	Zhang et al. (2022)
			2. Suppressing amyloid precursor protein	
	TRAP	25 μg/kg	1. Rescued the downregulation of MBP	Li et al. (2018)
			2. Improved remyelination	
Genetic Engineering	Knockout of LCN2	None	1. Attenuated myelin damage	Egashira et al. (2014), Toyota et al. (2019), Li et al. (2022)
			2. Attenuated oligodendrocyte loss	
			3. Oligodendrocyte precursor cell differentiation	
	Knockout of MMP-9	None	Improve myelin integrity	Egashira et al. (2015)
	PAR1 siRNA	None	Promoted OPC migration and remyelination	Li et al. (2018)
	Catalase CRISPR	None	1. Increasing the level of MBP	Xu et al. (2020)
			2. Decreasing the levels of APP, IL-6 and TNF-α	

First, in a previous study, endogenous proteins or molecules, such as heparin and melatonin, were administered. Intravenous infusion of undivided heparin (10 U/kg/h) into rats could significantly reduce myelin sheath injury, neuroinflammatory reactions and trans-synaptic neuronal apoptosis at 12 h after SAH (12). Afterwards, intraperitoneal injection of melatonin (50 mg/kg) after SAH could reduce the apoptosis of oligodendrocytes, the loss of MBP and the abnormal accumulation of APP by regulating the expression of Bim and Bcl-2 (16).

Second, when considering exogenous proteins or molecules (such as COG1410, RvD2 and TRAP) and after SAH, rats were intraperitoneally injected with the apolipoprotein apoE mimic peptide COG1410 (LPR1 ligand), which could significantly relieve myelin sheath injury, reduce brain water content, increase the M2 phenotype of microglia and improve neural function (Peng et al., 2019). Moreover, the administration of the G protein-coupled receptor 18 (GPR18) agonist RvD2 (resolvin D2) can relieve damage to the myelin sheath, reduce oxidative stress and apoptosis and protect the blood‒brain barrier by increasing the expression of MBP and inhibiting the expression of APP(17). Additionally, thrombin receptor antagonist peptide (TRAP, 25 μg/kg) can promote the regeneration of the damaged myelin sheath and the recovery of neural function after SAH (6).

Furthermore, the expression of proteins or molecules can be altered through genetic engineering. For example, the knockout of the *lcn2* gene in experimental animals can significantly reduce myelin sheath loss, axon injury, oligodendrocyte death and BBB damage after SAH (3, 7). When the *mmp-9* gene was knocked out, the T2 hyperintensity in the white matter of experimental mice was significantly lower than that of wild-type (WT) mice at 24 h after SAH; in addition, at 8 days after SAH, compared with *mmp-9* ^−/−^ mice, WT mice still had major myelin sheath injury (Egashira et al., 2015). Moreover, the transcription factor protease-activated receptor 1 (PAR1) is the key molecule in F-actin-dependent migration (Jeon et al., 2007). The downregulation of *par*1 expression with *par1* siRNA can promote myelin regeneration after SAH (6). Recent studies have also found that after SAH, the administration of catalase CRISPR (clustered regularly interspaced short palindromic repeats) to rats can also prominently increase the expression of myelin basic protein (MBP), while also reducing APP, IL-6 and TNF α and subsequently cricially alleviate the neurological deficit (Xu et al., 2020).

Generally, to treat myelin sheath injury after SAH, previous researchers have conducted many informative explorations from different aspects and obtained many important achievements, which have provided experimental conclusions and research experience for future precise treatments.

## 3 Deficiencies in the study of myelin sheath injury and treatment after SAH

Through the abovementioned analysis and summary, it can be easily deduced that the current research on myelin sheath injury after SAH is relatively superficial, and numerous problems have yet to be clarified. By comparing and analyzing the research results in adjacent fields, it has taken little effort to identify the shortcomings of current research. The shortcomings are detailed as follows.

### 3.1 Research on changes in individual characteristics of the myelin sheath

The individual characteristics of the myelin sheath (mainly including length, thickness, internode length and constituent proteins, etc.) are closely related to its function. Much research has been done on the individual characteristics of the myelin sheath, and considerable important progress has been obtained. As proof, the length of the myelin sheath is determined by the inherent characteristics of oligodendrocytes (Bechler et al., 2015; Swire et al., 2021); moreover, the main regulatory factor is tubulin polymerization promoting protein (TPPP) (Roll-Mecak, 2019), and the length of the myelin sheath in various areas of the central nervous system can be different (Hildebrand et al., 1993). The increase in myelin sheath thickness is a relatively independent stage in the maturation process of oligodendrocytes (Furusho et al., 2012; Kim et al., 2012), which is regulated by the surrounding axons, microglia and extracellular matrix proteins, among other factors (Friedrich and Mugnaini, 1983; Murcia-Belmonte et al., 2016; Nemes-Baran et al., 2020). The internodal length of the myelin sheath is also closely related to its surrounding microenvironment, which involves the number of OPCs, axon activity and extracellular matrix adhesion molecules, among other factors (Murcia-Belmonte et al., 2016; Hamilton et al., 2017; Osanai et al., 2018); however, it has nothing to do with the diameter of axons and the internal characteristics of oligodendrocytes. In addition, the main myelin sheath proteins are expressed and deposited in chronological order (Monge et al., 1986), and there are significant differences in the subcellular assembly positions of different myelin sheath proteins (Gould et al., 1999). These findings provide a basis for our comprehensive and profound understanding of the functional changes in the myelin sheath.

As mentioned above, SAH can obviously damage the individual characteristics of the myelin sheath. However, most of the relevant studies were limited to the influence on myelin sheath thickness and constituent proteins, and only a preliminary discussion had been made on the temporal and spatial changes of myelin sheath injury. Moreover, its pathogenic factors and pathophysiological mechanisms still need to be further clarified. Therefore, multiangle and multidimensional research on the changes in myelin sheath individual characteristics after SAH is helpful to clarify the relationship between the spatiotemporal changes in myelin sheath injury and its functional changes, as well as providing theoretical support and an experimental basis for the clinical treatment of myelin sheath injury and secondary neurological deficits.

### 3.2 Research on the pathophysiological mechanism of myelin sheath injury

**FIGURE 2:**
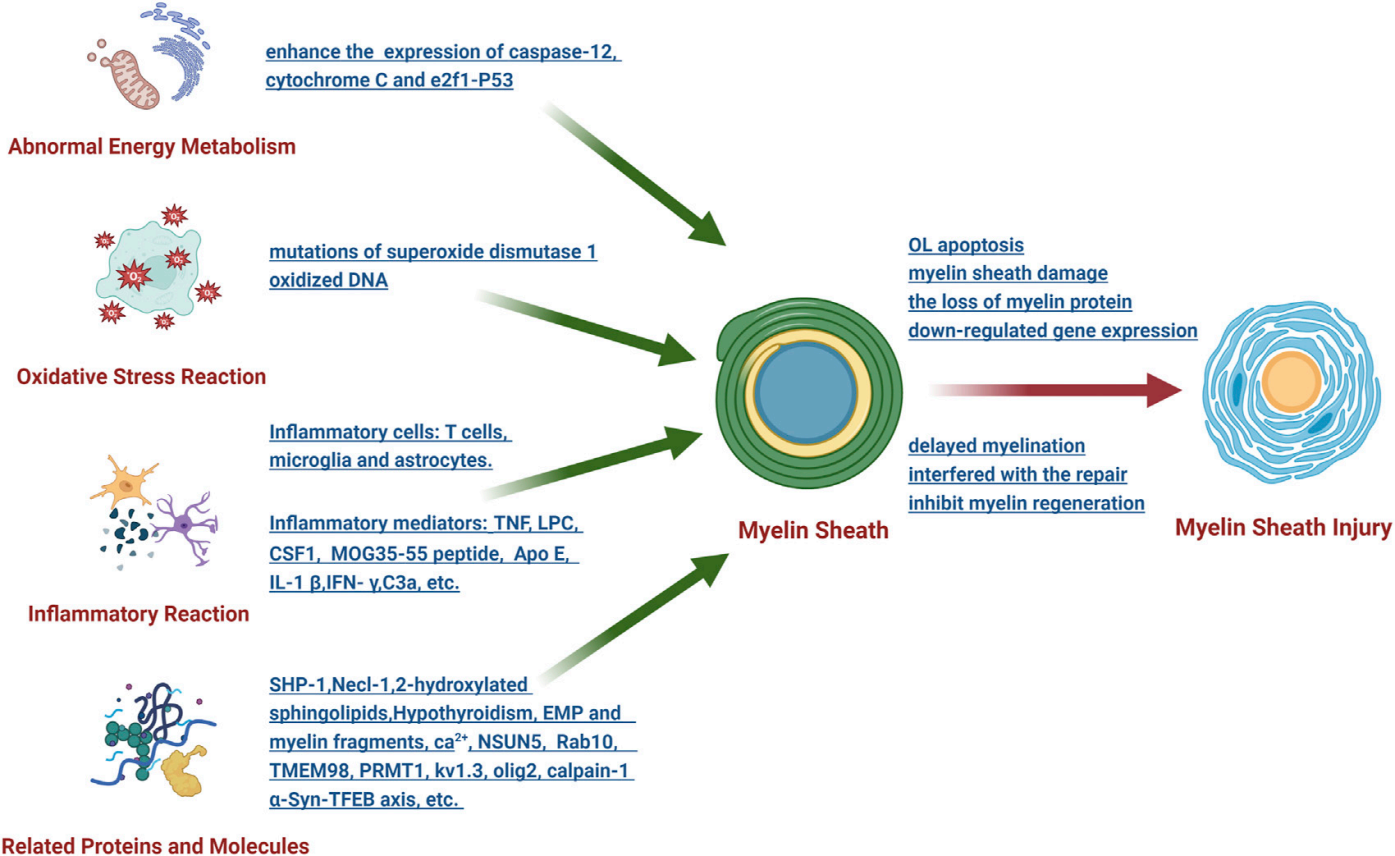
Schematic diagram of the research on the pathophysiological mechanism of myelin sheath injury after SAH.

Myelin sheath injury can occur in many central nervous system diseases, such as multiple sclerosis, nerve trauma, infection, cerebral hemorrhage and ischemic brain injury. Through in-depth analysis of the pathophysiological mechanism that causes myelin sheath injury, it was demonstrated that the mechanism mainly focused on the following aspects (Figure 2).

#### 3.2.1 Abnormal energy metabolism

The endoplasmic reticulum and mitochondria are the main participants in oligodendrocyte energy metabolism, which plays an important role in myelin sheath injury. To illustrate this point, after intracerebral hemorrhage, the levels of caspase-12 (a representative protein of endoplasmic reticulum stress) and cytochrome C (an apoptotic factor released by mitochondria) significantly increased, thus leading to oligodendrocyte apoptosis, which correspondingly results in demyelination (Zhuo et al., 2016). Compressive spinal cord injury can enhance the ER-mitochondrial interaction and the expression of e2f1 and initiate demyelination through p53-mediated oligodendrocyte apoptosis (Ma et al., 2017). As mentioned above, SAH can cause abnormal energy metabolism of the white matter, whereas the relationship between abnormal energy metabolism and myelin sheath damage has not been clarified.

#### 3.2.2 Oxidative stress reaction

After the central nervous system is injured, it is simple to produce oxidative stress reactions in its adjacent areas, and the myelin sheath is the most vulnerable structure. It has been demonstrated that mutations of superoxide dismutase 1 in amyotrophic lateral sclerosis can induce myelin sheath damage (Kim et al., 2019). However, the use of nano SIMS technology (nanoscale secondary ion mass spectrometry) demonstrated that oxidative stress can easily downregulate the expression of myelin sheath-related regulator genes through oxidized DNA, thus leading to axon damage and oligodendrocyte death (Giacci et al., 2018). At present, there is only one study on oxidative stress and myelin sheath injury after SAH, which is worthy of further study in the future.

#### 3.2.3 Inflammatory reaction

Inflammatory reactions are the most common pathogenesis, mainly involving inflammatory mediators and inflammatory cells. Research has shown that the inflammatory mediators that can cause myelin sheath damage mainly include TNF, LPC, CSF1, MOG35-55 peptide, Apo E, IL-1 β, IFN- γ and C3a, among others. The inflammatory cells involved in myelin sheath injury include T cells (Batterman et al., 2021), microglia and astrocytes. Inflammatory mediators and inflammatory cells lead to the loss of myelin protein, thus interfering with the repair of myelin sheath and ultimately affecting the structure and function of myelin sheath through their respective downstream pathways. Many studies have been performed on the relationship between inflammatory reactions and myelin sheath injury after SAH, whereas some research conclusions have been reported in other disease models for a long period of time, which deserves our vigilance and attention.

#### 3.2.4 Effects of related proteins and molecules

Previous studies have shown that many proteins and molecules can affect the formation, stability, maturation and regeneration of the myelin sheath. For example, SHP-1 is a key regulator of the myelin sheath development signal, and knockout of its gene can cause a myelin sheath formation disorder in mice (Massa et al., 2004). Moreover, knockout of the *necl-1* gene can destroy the initial recognition and adhesion between axons and oligodendrocytes, thus resulting in delayed myelination (Park et al., 2008). In the absence of 2-hydroxylated sphingolipids, a myelin sheath with normal structure and function can be formed, yet it cannot be maintained for a long period of time (Zoller et al., 2008). Additionally, hypothyroidism may cause the wrapping of the myelin sheath to axons to be obstructed. Extracellular matrix proteins and myelin fragments produced by demyelination can inhibit myelin regeneration (Baer et al., 2009; Bauch and Faissner, 2022). Other molecules, such as Ca^2+^, NSUN5, Rab10, TMEM98, PRMT1, kv1.3, olig2, calpain-1 and α-Syn-TFEB axis, among others, ccan also affect various aspects of the myelin sheath in different ways. As mentioned above, SAH can damage the myelin sheath through different proteins and molecules. Nevertheless, current research has mainly focused on clarifying its role and lacks in-depth discussion regarding its pathophysiological mechanism.

In brief, the pathophysiological mechanism of myelin sheath injury involves many factors and is very complex. At present, research on the mechanism of myelin sheath injury after SAH is associated with problems such as shallowness and lack of systematic methodology, and research has mainly focused only on oligodendrocytes. Whether there is a unique pathophysiological mechanism of myelin sheath injury is worthy of serious discussion.

### 3.3 Research on the treatment of myelin sheath injury

Myelin sheath injury has a significant impact on the integrity of nerve function; thus, the question of how to treat it is the focus and hotspot of current research. In general, the current research on the treatment of myelin sheath injury in adjacent fields has mainly focused on the following three directions.

#### 3.3.1 Seeking drugs to treat myelin sheath injury

From the perspective of clinical application, it is more convenient and practical to treat myelin sheath injury with drugs. Consequently, most studies have focused on this scenario. Overall, the research in this field can be divided into four facets (Table 2). The first aspect is to identify proteins or molecules that can accurately treat myelin sheath damage. For example, the GPR17 inhibitor pranlukast (Ou et al., 2016), GPR30-specific activator G1 (43), A2bAR selective activator BAY 60-6583 (44), TRPV4 antagonist Rn-1734 (45), LINGO-1 antagonist (Mi et al., 2007), estrogen receptor-β ligand (Crawford et al., 2010), Trk-b agonist Lm22a-4 (48), translocator protein PIGA 1138 (49) and monosialotetrahexosyl ganglioside (GM1) are involved in the regulation of lipid rafts (Zhang et al., 2011), and L-carnitine regulates the PTEN/Akt/mTOR signaling pathway (Ueno et al., 2015). The second facet is to search for proteins or molecules that can treat myelin sheath injury. For example, ethyl pyruvate (EP) (He et al., 2019; He et al., 2021), osteopontin (Selvaraju et al., 2004), structural analog of BDNF(55), nanoparticles containing leukemia inhibitory factor (Rittchen et al., 2015), clemastine (Li et al., 2015), endothelin 2 (58), human hair keratin (Xu et al., 2003), the cycloskeleton adapter protein anilin (ANLN) (Erwig et al., 2019), DL-3-n-butylphthalide (NBP) (Wu et al., 2019) and FMRP (62) can be used to treat this injury. The third aspect is to search for marketed drugs with therapeutic effects on myelin sheath injury, such as clobetasol (Su et al., 2020), trifluoperazine (Khaledi et al., 2021), sildenafil (Nunes et al., 2016), adrenomedullin (Maki et al., 2015), omega-3 polyunsaturated fatty acids (Pu et al., 2013), ascorbic acid (Nam et al., 2019), fluoxetine (Li et al., 2020a), melatonin (Abo Taleb and Alghamdi, 2020) and a mixture containing docosahexaenoic acid, arachidonic acid, vitamin B12, vitamin B9, iron and sphingomyelin (Hauser et al., 2020). The fourth facet is to investigate Chinese herbal medicine and its extract that can treat myelin sheath injury, such as Honokiol (Tan et al., 2022a), Astaxanthin (Lotfi et al., 2021), Shenling oral liquid (Liu et al., 2020b; Zheng et al., 2021), Rosmarinic acid (Li et al., 2020b), Naringenin (Rong et al., 2017), Salvianolic acid B (78), Tanshinone IIA (79), Bushen Yisui capsule (Rabinovich and Kacen, 2012; Zhao et al., 2018), the herbal medicine Ninjin ‘yoeito (Seiwa et al., 2007) and Quercetin (Naeimi et al., 2018; Tan et al., 2022b), among other medicines.

**TABLE 2:** Summary of therapeutic drug research on myelin sheath injury caused by other diseases.

Category	Name	Dosage	Intrinsic function	Main outcome	Reference
**Proteins or Molecules**	Pranlukast	0.1 mg/kg	Targeted inhibition of Gpr17	Promoted remyelination	Dubois et al. (2016)
	G1	10 µg/100 g	GPR30 specific activator	Promoted remyelination	Hirahara et al. (2013)
	BAY 60-6583	80 μg/kg	A2bar selective activator	Protected the myelin sheath from degeneration	Ma et al. (2022)
	RN-1734	10 µM	TRPV4 antagonist	Alleviated demyelination and inhibited glial activation	Liu et al. (2018)
	LINGO-1 antagonist	8 mg/kg	Loss of LINGO-1 function	Improved axonal integrity and newly formed myelin sheaths	Mi et al. (2007)
	Estrogen receptor beta ligand	8 mg/kg	Direct neuroprotective effect	Significant increase in myelin sheath thickness and axon transport	Crawford et al. (2010)
	LM22A-4	500 µM	Trk-b agonist	Promoted remyelination and increased myelin sheath thickness	Nguyen et al. (2019)
	PIGA 1138	15 mg/kg	The translocator protein ligand	Preserving myelin basic protein (MBP) expression	Tremolanti et al. (2022)
	Monosialotetrahexosyl ganglioside (GM1)	50 mg/kg	Improves neurofascin155 Association with lipid rafts	Prevent myelin sheath damage	Zhang et al. (2011)
	L-carnitine	600 mg/kg	Regulates the PTEN/Akt/mtor signaling pathway	Ameliorating oxidative stress and increasing oligodendrocyte myelination of axons	Ueno et al. (2015)
	Ethyl Pyruvate	20 mg/kg	Could convert astrocytes into myelinating cells	Promoted astrocytes to phagocytized myelin debris and effective myelin repair	He et al. (2021)
		10 mg/kg	Increased the phagocytosis of myelin debris by microglia	Enhances spontaneous remyelination	He et al. (2019)
	Osteopontin	3 μg/mL	Interactions with several Integrins	Regulated myelination and remyelination	Selvaraju et al. (2004)
	TDP6	40 μM	Selective targeting of trkb	Promoted remyelination	Fletcher et al. (2018)
	LIF-NP	300 μg/mL	Activated pstat-3 signaling	Increased myelin repair and thickness of myelin per axon	Rittchen et al. (2015)
	Clemastine	10 mg/kg	Increased mature ols and MBP	Enhancing remyelination	Li et al. (2015)
	Endothelin 2	10 ng/mL	Activated the ET-B receptor type B pathway	Promote myelin regeneration	Yuen et al. (2013)
**Proteins or Molecules**	Human hair keratin	Unknown	Promoted the proliferation and Differentiation of ols	Enhances myelin sheath rebuilding and repair	Xu et al. (2003)
	ANLN	Unknown	Facilitates septin assembly	Prevent pathological outfoldings of CNS myelin	Erwig et al. (2019)
	NBP	20 mg/kg	Decreasing activation of the NF-κB signaling pathway	Increased myelin density	Wu et al. (2019)
	FMRP	Unknown	Regulates production of MBP	Promotes myelin sheath growth	Doll et al. (2020)
**Marketed Drugs**	Clobetasol	5 mg/kg	Promoting OPC differentiation	Induced MBP expression and myelin sheath formation	Su et al. (2020)
	Trifluoperazine	2 mg/kg	Targeting Nrf2 and IKB	Reducing the demyelination	Khaledi et al. (2021)
	Sildenafil	25 mg/kg	Modulates inflammation	Contributes to myelin repair	Nunes et al. (2016)
	Adrenomedullin	40 nM	Via AM-receptor-PI3K/Akt Pathway	Increased myelin-basic-protein expressing	Maki et al. (2015)
	Omega-3 PUFAs	15 g/kg	Direct effect and suppression of inflammatory response	Prevented the loss of MBP and preserved the integrity of the myelin sheath	Pu et al. (2013)
	Ascorbic acid	100 mg/kg	Increased Olig2 and MAG expression	Promotes axonal myelination	Nam et al. (2019)
	Fluoxetine	10 mg/kg	Inhibition of rhoa/ROCK Pathway	Rescue myelin damage and reduce the expression of the negative regulatory protein of myelination	Li et al. (2020a)
	Melatonin	80 mg/kg	Antioxidant and anti-Inflammatory	Prevent demyelination and promote myelin regeneration	Abo Taleb and Alghamdi (2020)
	A blend containing docosahexaenoic acid, arachidonic acid, Vitamin B12, Vitamin B9, iron, sphingomyelin	1.5 μM	Increased the number of opcs and promoted their differentiation and maturation	Beneficial for myelination	Hauser et al. (2020)
		0.5 μM			
		0.001 μM			
		0.05 μM			
		1 μM			
		25 μM			
**Chinese Herbal Medicine**	Honokiol	20 mg/kg	Mediate endoplasmic reticulum (ER)-mitochondria pathway	Protect neural myelin sheat from demyelination	Tan et al. (2022a)
	Astaxanthin	3 mg/kg	Reduces oxidative stress	Inhibiting demyelination	Lotfi et al. (2021)
	Shenzhiling oral liquid	40%	Promoting PI3K/Akt-mtor Signaling pathway	Increased the expression of myelin sheath-related proteins (MBP, MOG and PLP)	Liu et al. (2020b)
		2.9 mL/kg	The activation of the PI3K/Akt-mtor signaling pathway	Promoting the expression of myelin proteins	Zheng et al. (2021)
	Rosmarinic acid	20 mg/kg	Ameliorates hypoxia/ischemia	Improving remyelination	Li et al. (2020b)
	Naringin	40 mg/kg	Through the β-catenin/GSK-3β signaling pathway	Promotes remyelination	Rong et al. (2017)
	Salvianolic acid B	20 mg/kg	Inhibiting apoptosis	Protect the myelin sheath	Zhu et al. (2016)
	Tanshinone IIA	50 mg/kg	Antioxidant and anti-Inflammatory actions	Increased both myelin sheath thickness	Feng et al. (2019)
	Bu Shen Yi Sui capsule	3.02 g/kg	Alleviated inflammatory Infiltration	Protected the ultrastructural integrity of myelin sheath	Zhao et al. (2018)
			Via exosome-mediated Molecular signals	Protected the ultrastructural integrity of the myelin sheath and significantly increased the expression of MBP	Zhao et al. (2020)
	Ninjin’yoeito	1%	Restoration of fcr gamma/Fyn signaling	Repairs central nervous system demyelination	Seiwa et al. (2007)
	Quercetin	60 mg/kg	Regulating microglial Phenotype transformation	Reduced demyelination	Tan et al. (2022b)
		50 mg/kg	Alleviated the glial activation	Decreased the extent of demyelination areas and increased the remyelination process	Naeimi et al. (2018)

As mentioned above, there have been relatively few studies on the treatment of myelin sheath injury after SAH. The search for effective drugs to treat myelin sheath injury from the abovementioned four aspects has indicated the research direction and broadened the research ideas.

#### 3.3.2 Cell transplantation or genetic engineering to treat myelin sheath injury

It is also an important research direction to treat myelin sheath injury through cell transplantation or genetic engineering. With regard to cell transplantation, umbilical cord mesenchymal stem cells (Zhang et al., 2012; Chen et al., 2013), neural stem cells (Hwang et al., 2009) and f3. olig2 cells (Ahn et al., 2016) can promote myelin sheath repair and improve neural function. Genetic engineering, such as knockout of the *G-protein coupled receptor-17 (GPR17)* gene, *GPR62* gene, *hnRNP-K* gene, *EPAC* gene and *Lingo-1* gene, as well as overexpression of the *Golli myelin basic protein* gene, *SOX10* gene and *NF155* gene and regulation of *FGF receptor* gene expression (Li and Yao, 2013), can effectively treat myelin sheath injury. Although this research direction has experienced many difficulties in clinical application, it is still worthy of further discussion.

#### 3.3.3 Attempts of physical therapy to treat myelin sheath injury

It has been proven that physical therapy can treat myelin sheath injury in experimental animals. A previous study demonstrated that precisely timed motor learning can promote the recovery of myelin sheath injury by enhancing the remyelination of newborn and surviving oligodendrocytes (Bacmeister et al., 2020). The application of electroacupuncture can promote the proliferation of oligodendrocytes and inhibit their death to mediate the protection of the myelin sheath (Huang et al., 2015). Moreover, electroacupuncture treatment can also reduce myelin sheath fragments, promote microglia and oligodendrocytes to gather to the injured site, upregulate the expression of MBP and AXL in the corpus callosum of demyelinating mice and accelerate myelin sheath regeneration (Kang et al., 2020). These pioneering studies have provided us with new ideas and methods for the clinical treatment of myelin sheath injury after SAH, which is worthy of continuous attention and trial application.

In general, the current research on myelin sheath injury and treatment after SAH has experienced some problems, such as relatively superficial research, ineffective connection between basic research and clinical application and no effective drugs entering clinical application. Knowledge concerning the treatment strategies and achievements in adjacent research fields can provide new ideas for research and new methods for the precise clinical treatment of myelin sheath injury.

## 4 Perspective

The early diagnosis and treatment of myelin sheath injury after SAH is of great significance. Based on the spatiotemporal change characteristics of myelin sheath injury, the clarification of the pathophysiological mechanism and precise treatment is a practical research approach. In view of this research idea, the following points warrant further discussion.

### 4.1 Detection of the spatiotemporal change characteristics of myelin sheath injury

The spatiotemporal change characteristics of myelin sheath injury were the basis of treatment research after SAH. Therefore, how to quickly, accurately and comprehensively detect this change is of profound significance. In view of the complexity of the spatiotemporal change characteristics of myelin sheath injury after SAH, the information provided by a single technical method is limited. Hence, multimodal detection combined with neuroimaging, neuroelectrophysiology, electroencephalography, real-time metabonomics and other technologies can allow for the possibility to understand the spatiotemporal change characteristics of myelin sheath injury in a multidimensional, multiangle and systematic manner after SAH. It is possible that neuroimaging can be used to assess the location and extent of myelin sheath injury. As mentioned above, T2-hyperintensity of MRI in white matter is closely related to degradation of MBP after SAH (3). The real-time detection of metabolic biomarkers in the blood, cerebrospinal fluid, tears, saliva, urine and other body fluid samples can accurately reflect the changes in the myelin sheath, which has provided researchers with the possibility to accurately describe the occurrence, development and outcome of myelin sheath injury after SAH. Spatial metabolomics based on high spatial resolution imaging mass spectrometry can visually assess the composition, dynamics and spatial distribution of protein molecules in tissues or cells (Chen et al., 2022a; Chen et al., 2022b). In addition, traditional examination methods such as neuroelectrophysiology and electroencephalography still have value for in-depth research on the continuous monitoring of myelin sheath injury due to their convenience, low cost and high popularity. Consequently, by combining different inspection methods, real-time multimodal detection can be helpful to accurately clarify and monitor the spatiotemporal change characteristics of myelin sheath injury after SAH and can provide experimental data and a theoretical basis for treatment research.

### 4.2 Screening of research objects for treatment of myelin sheath injury

Nonetheless, various important research achievements have been made in the treatment of myelin sheath injury after SAH in recent years; however precise treatment still has not been achieved. The main reason involves the deviation in the screening of research objects. SAH can cause myelin sheath injury with many pathogenic factors, complex pathophysiological mechanisms and extensive structural and functional damage. Consequently, it is believed that the research object for the treatment of myelin sheath injury should focus on the starting point of SAH, the intersection point of multiple pathophysiological mechanisms and the joint event leading to different myelin sheath structures and functional damage. Via single-cell sequencing, spatial real-time metabonomics, comparative proteomics and other methods, key research objects have been identified, after which drug molecules (preferably with multiple action targets) were screened to finally achieve precise treatment of myelin sheath injury after SAH. Moreover, it is believed that the treatment of myelin sheath injury should not only focus on the myelin sheath but also focus on how to restore the balance of cerebrospinal fluid circulation and the reconstruction of the intracranial barrier structure. Hence, it is necessary to actively explore other treatment methods and study the mechanism of action, such as the abovementioned electroacupuncture treatment.

## 5 Conclusion

Although research on the diagnosis and treatment of myelin sheath injury after SAH has made many significant achievements, a number of problems remain. Based on the spatiotemporal change characteristics of myelin sheath injury after SAH, a focus on the starting point, intersection point and common event, as well as devoting attention to the overall situation and actively exploring different treatment methods, may result in new breakthroughs for its diagnosis and treatment.
